# Patient research priority setting partnership in human T‐cell lymphotropic virus type I

**DOI:** 10.1111/hex.13848

**Published:** 2023-08-14

**Authors:** Adine Adonis, Anne‐Marie Russell, Graham P. Taylor, Melanie Preston, Alpheus Shields, Sue Strachan, Sky Young, Haissata Diallo, Stephen Ashford, Elizabeth Cassidy

**Affiliations:** ^1^ National Centre for Human Retrovirology, Imperial College Healthcare NHS Trust London UK; ^2^ Institute of Health Research University of Exeter Exeter UK; ^3^ Imperial College London, Faculty of Medicine London UK; ^4^ Patient Collaborators London UK; ^5^ The Regional Hyper‑Acute Rehabilitation Unit Northwick Park Hospital, London North West University Healthcare NHS Trust London UK; ^6^ Department of Palliative Care, Policy and Rehabilitation The Cicely Saunders Institute, King's College London London UK; ^7^ Freelance Qualitative Researcher

**Keywords:** collaboration, communication, HTLV‐1, priority setting, research needs, user involvement

## Abstract

**Introduction:**

Human T‐cell lymphotropic virus type 1 (HTLV‐1) is a chronic infection affecting 5–10 million people worldwide. Ten percent develop HTLV‐1‐associated diseases, and 3%–5% develop HTLV‐1‐associated myelopathy (HAM)/tropical spastic paraparesis. Low health‐related quality of life (HRQoL) is a significant concern for those with HTLV‐1, and little is known about how it impacts daily life or what patients need from healthcare services. To address this, we report on patient involvement workshops aimed at identifying research priorities for HTLV‐1 health service provision.

**Methods:**

Participants recruited through HTLV‐1 clinics in England attended six 90‐min virtual workshops over 10 months, and two 60‐min consolidation workshops. Content developed iteratively from topic focussed group discussions. All workshops were video‐recorded with consent, transcribed verbatim and thematically analysed. Using consensus voting rounds, participants individually ranked their top six and then collectively their top three research priorities from the themes inferred from the analysis. A final feedback session explored the experiences of participating in the workshops.

**Findings:**

Twenty‐seven people with HTLV‐1 engaged with the workshops with up to 22 participants attending each meeting. The majority were diagnosed with HAM (*n* = 22). The top three research priorities were identified as understanding disease progression, psychosocial wellbeing, and information and knowledge. Participants valued being asked to set research priorities that directly addressed their needs and enjoyed the workshops. They stressed the importance of patient advocates for promoting research that positively impacts everyday life.

**Conclusion:**

This is the first of this type of research engagement with people with HTLV‐1 in the United Kingdom. Participants identified several avenues of investigation that could lead to improvements in healthcare services and HRQoL. Participants believed the workshops signified the start of a conversation to progress person‐centred and meaningful research in HTLV‐1.

**Patient or Public Contribution:**

People living with HTLV‐1 were involved in the iterative design, conduct, analysis, writing and dissemination of this project through the patient involvement workshops. As a result of this engagement, a patient led advisory group has been set up to assist with the dissemination of the findings.

## 1 BACKGROUND

There is no cure or vaccine for human T‐cell lymphotropic virus type 1 (HTLV‐1). In England and Wales, approximately 22,000 people live with HTLV‐1,^1^ the majority are women (65%), of Black Caribbean ethnicity (60%) and born outside the United Kingdom (72%).^2^ This underserved community of people living with a rare condition were empowered to identify their research priorities.

HTLV‐1 is a retrovirus affecting an estimated 5–10 million people worldwide.^3^ The virus is transmitted via contaminated blood products (including from blood transfusions, organ transplants, and injection drug use), sexual contact, and from mother to child via breastfeeding.^4^ High levels of infection are clustered in certain geographical regions, predominantly Southern Japan, South America, sub‐Saharan Africa, and the Caribbean.^3^

Most people infected with HTLV‐1 have no known HTLV‐1‐related health conditions and are described as asymptomatic carriers. Nonetheless, asymptomatic HTLV‐1 is associated with reduced health‐related quality of life (HRQoL)^5^ and a wide range of symptoms have been reported including pain and discomfort,^5^, ^6^, ^7^ bladder and visual disorders, neurological and rheumatological symptoms, and dermatological and oral health conditions.^8^ It is unclear whether these symptoms signify the early onset of an intermediate syndrome, HTLV‐1‐associated myelopathy/tropical spastic paraparesis (HAM/TSP)^8^ or an oligosymptomatic group.^9^

Symptomatic HTLV‐1 infection is linked with the onset of inflammatory conditions, such as uveitis, infective dermatitis and polyarthritis and two rare, severe health conditions, HAM/TSP and adult T‐cell leukaemia/lymphoma (ATLL).^4^

## 2 HAM/TSP

The lifetime risk of developing HAM/TSP varies across infected communities, with estimates between 0.25% and 3.7%.^10^, ^11^ It is more common in women, with a female‐to‐male ratio of 2:1–4:1.^12^ HAM/TSP is associated with HTLV‐1‐mediated inflammation of the spinal cord causing long‐term progressive neurological degeneration leading to spastic paraparesis with muscle weakness and stiffness, bladder and bowel dysfunction, and sensory disturbances of the lower limbs.^12^ Moderate to severe pain is a persistent and common problem, affecting approximately 90% of people with HAM/TSP.^6^, ^7^ Symptoms are most commonly slowly progressive and usually develop in midlife. Mobility declines over a period of approximately 20 years, during which walking aids or wheelchairs usually become necessary for everyday function.^12^, ^13^ Rapid progression over weeks is seen in a subset. People with HAM/TSP have an extremely low HRQoL and record lower HRQoL scores than those of people with a range of health conditions including multiple sclerosis, epilepsy and diabetes.^5^

Treatment of HAM/TSP is challenging as there is no cure. Clinical care is focused on symptomatic management of spasticity, pain, and bladder and bowel problems.^12^ Physiotherapy may have a positive impact on impairments, disability, participation and quality of life.^14^, ^15^ A broad range of potentially disease‐modifying therapies have been tried, usually in observational studies, and the best results to date have been with corticosteroids, which may improve pain and motor function acutely and slow disease progression over the long term.^12^, ^16^

## 3 ATLL

ATLL is an aggressive cancer with a poor prognosis and limited treatment options.^17^ HTLV‐1 carriers have a 3%–5% lifetime risk of developing ATLL.^18^ Care for people with ATLL in England is informed by and shared across HTLV‐1 and cancer services and follows the national clinical guidelines for blood cancer services.^19^

## 4 HEALTHCARE APPROACH

In England, the national referral centre for HTLV‐1 and specialist multidisciplinary outpatient service uses a hub and spoke model, with a highly specialist centre in London (hub) and three satellite centres in the Midlands and the north of England (spoke). Referrals under different public health payment models are also accepted from clinicians in Wales, Scotland, and Northern Ireland. This care pathway has been in place for just under 20 years and includes physiotherapists, clinical nurse specialists, and consultant physicians in sexual health, infectious diseases, neurology, human retrovirology, and haematology. Psychological support is provided through referral to external services. The diagnostic and follow‐up support offered through the national HTLV‐1 service may in part account for the lower levels of anxiety and depression reported by people with HTLV‐1 in the United Kingdom compared to those in Brazil where similar services are not available.^5^

## 5 CURRENT LANDSCAPE OF HTLV‐1

Despite decades of research which has enhanced the scientific understanding of HTLV‐1, most healthcare professionals have very little knowledge of this condition or its impact.^20^ Qualitative research undertaken in Brazil has shown that people with HTLV‐1 often feel invisible and neglected by a healthcare system that takes a predominately biomedical approach.^21^ While much attention is given to diagnosis, medical and risk management, the impact of emotionally painful experiences such as stigma, social judgement and exclusion, as well as the financial hardship, dependency, loss of roles and productivity, and impaired relationships that may accompany a diagnosis of HTLV‐1 are often overlooked.^21^, ^22^ Socialisation, comfort in the face of adversity and new meanings for life and suffering have been found through spiritual and religious practices, or meditation.^23^ Exercise improves socialisation and autonomy but may be hampered by financial problems and limited access to appropriate facilities.^22^ Little is known about the subjective experience of living with HTLV‐1 in the United Kingdom and what people with this condition need from national and local healthcare services.

## 6 AIM

We set out to identify research priorities from the perspective of people with HTLV‐1. We ran a series of patient involvement and engagement workshops to explore participants' perspectives about living with HTLV‐1, and the strengths and limitations of their interactions with healthcare professionals and services. We were interested to understand how the current healthcare pathway addressed the needs of people living with HTLV‐1 from their perspectives.

## 7 METHODS

### 7.1 Patient engagement in research

Including patients in research is important to ensure that the research is relevant, ethical, feasible, and accessible.^24^ Patients and the public in the United Kingdom have the right to participate in the design, management, conduct, and dissemination of research, and researchers should strive to meet UK standards for public involvement in research.^25^, ^26^, ^27^ People living with HTLV‐1 are considered an underserved group as they share several characteristics with those who should receive better service from the research community. They have limited opportunities to participate in research, a high healthcare burden, unmatched by the amount of research undertaken for the group, unexamined and important differences in how a group responds to or engages with healthcare interventions compared to other groups, and limited understanding of how to optimise healthcare engagement and outcomes.^28^

We remained true to the NIHR definition of public involvement in healthcare research,^29^ in planning and running the patient involvement workshops, and by ensuring that people with relevant lived experiences were actively involved at the beginning of the design and development stages of our research process. All activities were undertaken as a collaboration between people with HTLV‐1, clinicians, and researchers. We used an inclusive approach following the UK Standards for Public Involvement.^27^

### 7.2 Recruitment

Adults, aged 18 years or over, living with HTLV‐1, either as asymptomatic carriers or with HAM/TSP, and registered with one of the specialist multidisciplinary HTLV‐1 clinics in England, were invited to participate in a series of virtual (online) patient involvement workshops. Flyers were handed out at outpatient clinics and displayed in the clinic waiting room over the course of 3 months. The flyers (Supporting Information) explained the purpose of the workshops and the meeting format. The lead author (A. A.), a clinical specialist physiotherapist in HTLV‐1 and neurology at the National Centre for Human Retrovirology, UK, described the aims of the workshops in more detail to those who expressed an interest, and answered questions, either face‐to‐face in clinic or by telephone. All who expressed an interest were invited to the first online meeting and encouraged to invite people from their networks to attend. Adults with ATLL follow an established blood cancer care pathway,^19^ and have a different illness trajectory compared to those who are asymptomatic and those with HAM/TSP and were therefore excluded.

### 7.3 Procedure

Six online workshops were held from January to October 2021. Each workshop lasted 60–90 min. Participants were not obliged to attend all the workshops. Everyone received post‐event information via email which included summaries and questions to consider in advance of the next meeting. Two further consolidation online workshops were held in December 2021 and March 2022 to share progress with grant applications and update participants on further research opportunities.

All workshops were delivered online. Participants attended via laptops, tablets, or smart phones. A. A. provided digital support enabling participants to connect and download the meeting platform and familiarised them with the user interface. An email reminder of the date, link and meeting agenda was sent out to all participants 2 weeks before each meeting. One week before each meeting, participants received a telephone call to remind them of the meeting. Participants who missed meetings were offered individual catch‐up sessions either in clinic or via a phone call with A. A.

The workshops and consolidation sessions were facilitated by A. A. and cofacilitated by a mixed methodologist with qualitative clinical research experience and rare disease specialism (A. M. R.) and a qualitative health researcher with a background in physiotherapy and research experience with people with rare neurological conditions (E. C.). All workshops were audio‐visually recorded with the permission of all attendees. Recordings were transcribed verbatim, anonymised, and stored securely on an encrypted drive in a secure office.

Each workshop (summarised in Table 1) had five components (a) introductions and socialisation, (b) summary of the aims and purpose of the workshops, (c) focused conversations on agreed topics with break‐out rooms where necessary to enable deeper discussion of selected topics, (d) summary and agreement of the next steps, (e) raising awareness of HTLV‐1‐related events and new involvement and engagement opportunities. The findings of a preliminary bespoke patient and public involvement (PPI) meeting, with a small group of people with HTLV‐1, conducted in 2019 were used as stimulus material for the workshop discussions. This preliminary meeting had gauged interest in involvement in research and identified clinical care priorities.

**Table 1:** Workshop schedule and content.

Workshop	Content
All sessions	Welcome and introductions. Ground rules for online workshops, confidentiality, terms of reference.
1 Introduction (January 2021)	Overview and aims of the session. The road map for the six workshops. Agreement of the number and frequency of meetings, dates, times, duration. *Q&A:* COVID vaccination and HTLV‐1.
2 Review of the 2019 PPI findings (March 2021)	*Focused conversation:* Discussion of the main findings from the 2019 PPI. ▪ Relevance of 2019 PPI findings in 2021. ▪ What's missing? ▪ What do you find difficult about living with HTLV‐1? ▪ What issues do you want to focus on? ▪ What do you need from healthcare providers? ▪ How can healthcare providers and services address the problems you describe? *Engagement opportunity:* WHO online launch of the HTLV‐1 technical report.
3 Drilling down: Living with HTLV‐1 (May 2021)	*Focussed conversation:* Breakout rooms used for small group discussion. ▪ What are your experiences of living with HTLV‐1 as an asymptomatic carrier? What do you find difficult about living with HTLV‐1? ▪ What are your experiences of living with HTLV‐1 as someone with HTLV‐associated myelopathy? What do you find difficult about living with HTLV‐1? ▪ Whole group feedback and discussion. *Postworkshop activity:* Participants were asked to select six priority topics from a list of 13 topics derived from the discussion to discuss at the next meeting.
4 Prioritising topics (June 2021)	*Focussed conversation:* Discussion of six prioritised topics, breakout rooms to discuss different topics (agreements and disagreements) in more depth. *postworkshop activity:* participants were asked to select three priority topics.
5 Agreement of top three priority topics (August 2021)	*Focussed conversation:* ▪ Rank ordering prioritised topics ▪ Voting on the top three topics ▪ Discussion in breakout rooms of the top three topics
6 Feedback and review (October 2021)	Facilitated reflection and review: ▪ Experience of participating in the workshops. ▪ Strengths and limitations.

Abbreviations: HTLV‐1, human T‐cell lymphotropic virus; PPI, patient and public involvement; WHO, World Health Organization.

The sixth workshop, a feedback session, was facilitated by experts in Public and Patient Involvement and Engagement from Imperial College London who had not been involved in the delivery of the previous workshops. The feedback session allowed participants to talk freely about their involvement in the workshops and to reflect on its strengths and limitations. Questions were sent in advance (Table 2) and participants were invited to submit responses via email if they were unable to attend live (no email submissions were received).

**Table 2:** Workshop feedback session questions.

Questions	Supplementary questions
In your opinion, how do you think the series of workshops went?	What was most memorable/different/interesting. Were there any turning points/critical points in these sessions for you. What didn't happen that you had thought/hoped would happen? What did you like about the experience?
Where could more time have been spent?	Where could less time have been spent?
How do you feel about this piece of work?	What are the most important things that you have takeaway from the session? What expectations did you have about this work? Were your expectations met? What has changed for you since participating in these sessions?
What do you think the next steps should look like?	Where are you hoping this work leads to? Would it be helpful to tell others about this work?
If you had the opportunity to be involved in something similar in the future, would you consider participating?	
If you could do it all over again, what would you change?	What is the one thing that could have been improved on in these workshop series?

### 7.4 Iterative data collection and data analysis

Recordings of the first five workshops were transcribed verbatim and anonymised by A. A. The transcripts were independently thematically analysed by A. A., E. C. and A. M. R. identifying patterns, similarities, and differences in the data according to the topics addressed in the second and third workshops (Table 1). This stage of the analysis was guided by the principles of qualitative thematic data analysis.^30^, ^31^ Critical discussion of the proposed themes with the co‐facilitators helped to consolidate ideas and prompt further analysis and reflection. Thirteen potential research topics were inferred from the analysis (Table 3).

**Table 3:** Initial research priorities and exemplar quotations.

S. no.	Theme	Exemplar quotations
1	Diagnosis	‘…can't get my head round it really, all of a sudden, got this disease and my life has completely changed. I can't do the things I were able to do’. ‘…my biggest question is, how long have I had this condition? Where did I get it?’ ‘…when I was initially diagnosed […] that sort of fear of what's going to happen? How will you end up? Are you going to be disabled? Or you're going to have to rely on people and really never walk again? And just the not knowing. It did change my life’.
2	Disease progression	‘I'm not sure where I'm at with the progression of the disease. I mean, when I come to you guys for my check‐up… and I don't know if I'm stable or if I'm declining’. ‘If my condition should progress, what am I looking out for? What are the changes? How do you know? How does it manifest itself? So it's quite, it's quite frightening in a way because of the unknown’. ‘I think you still tend to feel that you're just getting worse even though the virus is not multiplying in your body, so to speak. Because this thing kind of creeps up to you. Because you find one day, you're doing loads of things. And then the next day, you can't’. ‘That flat surface in the clinic […] doesn't reflect really, you know, our ability to walk because the, you know, in the hospital, it's different, […] to maybe when you're outside, so it doesn't reflect it really on how well we are able to walk’. ‘What do I look out for? How do I know if my disease is developing? Nobody's ever told me that what to look out for’.
3	Mental health	‘…it has affected my mental health … I've got the “Why me days”, you know, where you just want to sit in a dark room and think, well, why you know, of all, you know, the possibilities. Why is this happened to me? […] So I do struggle, every now and again with that when I'm having my low moments’. ‘I try to talk to my family as much as I can, I have church […]. We have our prayer meetings. We have Bible studies, there's all the things for me to do. So, because I'm busy like that, I don't find that I'm having time to think about mental issues and problems’.
4	Family/carer burden	‘… my frustration actually affects people around me. Because I can tell that they said, “Oh, just don't worry”. And I do worry about it because I just don't feel I should put any pressure on others or … expect too much from others”. ‘I've accepted that it's permanent. However, my friends and families haven't, which some people might think is a good thing. But sometimes it's a bit, it's a bit emotionally draining, they're, they're constantly trying to say to me that I'm going to beat this, just to boost my confidence’.
5	Psychosocial impact	‘If I'm not comfortable, and people are not accommodating, it makes you feel like …, no longer live the life you used to be. Now you live in this strange life …, living somebody else's life? Yeah, then you kind of give up a little bit’. ‘it's harder when you've gone to visit people […] all of a sudden, you need a toilet. You can stand up, you can go to the toilet, sit on the toilet and you can't stand up. No, you have to call someone. And they are busy talking and you stop that’.
6	Symptoms	‘I really get tired, and my legs get really weak if I go any distance. And I mean just go walking 500 yards down the road and my legs want to give out and I find that can be frustrating, tiring and yeah… gets me on mental stress. I just, I just get upset with myself not being able to, to walk very far’. ‘… the numbness and the stiffness that's in my body from my, from my waist all the way down… And then there's an altered sensation of hot and cold. You know, night‐time in the bed. My legs are so hot. The twittering in the daytime like I'm sitting now. My legs are so cold. I'm wrapped up in the blanket’. ‘I have continuous pains. I have difficulties walking. I have recurrent infections. I have difficulties passing urine. And also, I have difficulties opening my bowels’.
7	Healthcare professionals	‘… the medical care people, the doctors, nurses, etc., they need to be educated on the condition because when I went to hospital […] the nurse didn't seem to know what HTLV was’. ‘…the professional doesn't know what it is. So, they really need to, you know, get a little bit more to grips what this HTLV is. When an HTLV person referred to them, they ought to know a bit about it before’.
8	Accessing information and knowledge	‘Are there more people who are actually being diagnosed with this condition? Is it on the increase? And if it is it, what are the age groups? Or is it still affecting, say, the age group that we are in you know the over 40s or 50s upwards?’ ‘I wonder though, I have the virus, is it mild or is it like a trace or something like that? You know, I just don't know’. ‘It affects us greatly, mentally, physically, in all aspects of your life. HTLV does affect and there is no mention for it. You need to go and do research about it. And there is not much information. Google is a no no … it just makes you more worried’.
9	Lack of integrated services	‘… every one of my systems is affected, which means I see a different consultant, a different hospital for everyone’. ‘… when I go to my GP, it's very difficult to be seen as a whole. They don't connect all the dots. […] they just see the urine infection, they don't see you as a person’.
10	Importance of research	‘… research is very important for us because as far as I know, a treatment for HTLV one associated diseases are experimental’. ‘I wish that all patients are told about the ongoing researches and given the choices for trials on, on a continuous basis’.
11	Community	‘… certainly around volunteering, that would be something that I'd be extremely interested in offering support to people with the diagnosis around maximising their benefits and incomes and accessing care and support and stuff for the future’. ‘I'd like to see the HTLV one patient forum resume, but not in any previous form… like this group meeting, to be a place where patients can meet to share experiences, and concerns, etc. Also, I'd like it to be a group with a purpose, where we can focus on discussions to bring solutions to our problems’.
12	Treatment	‘… being on the steroids […] I wasn't a high dose. I've come down to a lower dose, but kind of the low dose has made me feel that I've gotten backwards. But I don't want to increase. I'm taking tablets, tablets and tablets. So therefore, I'm fighting with the devil. At the same time, of course, I'm in pain’.
13	Management including exercise	‘I find if I if I do a lot of exercise, I get really tired. Yeah, it seemed to make me worse rather than better’.

Abbreviations: GP, general practitioner; HTLV, human T‐cell lymphotropic virus.

Participants were invited to rank order their top six priorities from this list. Comparisons were made between the rank ordering of those with HAM/TSP and those who were asymptomatic carriers and presented to the group, in preparation for the fourth workshop. After further discussion of the individual and collective rank ordering of topics in the fourth workshop, participants were asked collectively to identify their top three prioritised topics. Participants voted on the final top three topics in the fifth workshop. The final selection of topics was achieved by consensus voting rounds.

The three priority topics were developed into draft research priorities and shared with the group at the consolidation workshop in December 2021. Following refinement based on PPI group feedback, the final prioritised research topics were shared in the consolidation workshop in March 2022.

Recordings of the feedback session (Workshop 6) were transcribed and anonymised by the facilitators. The transcript was reviewed by A. A., E. C. and A. M. R. for common themes concerning participation and participants' perceptions of the strengths and limitations of the workshops.

## 8 RESULTS

Thirty‐five people expressed an interest in participating. Twenty‐seven people with HTLV‐1 (HAM/TSP or asymptomatic carriers) engaged with the workshops. Up to 22 people attended each workshop. All participants were known to the lead author (A. A.) through her clinical role at the specialist centre for HTLV‐1. None of the participants were known to the co‐facilitators. All sessions were attended by participants diagnosed with HAM/TSP (*n* = 22), and asymptomatic carriers (*n* = 5). Participants formed a geographically and ethnically diverse group, ranging in age from 42 to 78 years old, with women (*n* = 20) the most highly represented group.

The three topics identified by the group as priorities for further research were: (a) understanding disease progression, (b) psychosocial wellbeing, and (c) information and knowledge. Each topic is presented below using exemplar quotations.

### 8.1 Disease progression

*“I think you still tend to feel that you're just getting worse even though the virus is not multiplying in your body, so to speak. Because this thing kind of creeps up to you. Because you find one day, you're doing loads of things. And then the next day, you can't.”*

The priority‐setting element of the workshops identified most participants were concerned about disease progression and were uncertain about its measurement in clinical practice. Participants found it difficult to understand the meaning of data derived from conventional biological testing (e.g. measures of HTLV‐1 proviral load) particularly when decisions about the presence or absence of disease progression were not aligned with how participants felt on a day‐to‐day basis or with the emergence of potentially new HAM/TSP symptoms. Participants struggled to assign meaning to new symptoms and changes in functional capacity not explained by changes in biological markers. Functional measures such as the 10m walk test were considered to have little ecological validity, lacking relevance to the participants' performance in the real world. An over‐reliance on biomedical measurement and management strengthened participants' perspectives that they were not ‘fully seen’ by healthcare practitioners and their concerns and fears were not adequately addressed.

### 8.2 Psychosocial wellbeing

*“Now you live in this strange life …, living somebody else's life? Yeah, then you kind of give up a little bit.”*

Participants prioritised further investigation of the psychosocial aspects of a HTLV‐1 diagnosis and its impact on their identity and emotional well‐being. They spoke powerfully about their struggle to get a diagnosis, the fear and anxiety that accompanied diagnosis, the lack of knowledge of HTLV‐1 shown by healthcare professionals and the unfamiliar and unsettling medical terminology they encountered in their visits to the clinic. Participants agreed that the difficulties and complexities of living their lives following their diagnosis were poorly understood by healthcare practitioners. Lack of specific mental health support and access to others with similar experiences were felt to compound their sense of isolation and invisibility.

### 8.3 Information and knowledge

*“It affects us greatly, mentally, physically, in all aspects of your life. HTLV does affect and there is no mention for it. You need to go and do research about it. And there is not much information.”*

The third priority focussed on the lack of knowledge of HTLV‐1 in healthcare and in the wider social world, and the need to raise awareness of HTLV‐1 to improve, not only healthcare management, but the public understanding of this condition too. Participants were concerned about what they perceived as a lack of evidence about the effectiveness of pharmacological interventions used to control disease progression and to treat symptoms of HAM/TSP. Finding accessible and reliable sources of information was difficult. Participants also questioned the role and value of exercise in managing symptoms and disability. Participants strongly advocated for more research and knowledge generation and for their continued involvement in shaping future research.

### 8.4 Findings of the feedback session (Workshop 6)

Participants valued the opportunity to take part in the workshops. They enjoyed the sense of community that meeting regularly over several months offered. Participants enjoyed and valued the camaraderie of meeting with and talking to others in a similar situation. They would have appreciated more time being afforded to socialisation and listening to each other's health and life stories. Participants felt galvanised by having a sense of purpose and for being trusted with and having the responsibility of setting research priorities. Participants appreciated that their individual and collective voices were able to advocate for research that directly addressed their needs. They expressed hope that their participation in these workshops would lead to more research being done in the areas they had highlighted were important.

## 9 OUTCOMES

The outcomes of these priority‐setting workshops have informed a subsequent research proposal and funding applications. Participants have joined the advisory group for this proposed study. Several spin‐off projects were identified and are being developed with workshop participants. At the request of participants, the group has been informed about additional involvement and engagement opportunities. Three participants were involved in presenting this project at an international conference.

## 10 DISCUSSION

A representative group of participants living with a diagnosis of HTLV‐1 attended six facilitated priority‐setting workshops over a period of 10 months. Thirteen research priorities were identified. The top three priorities were agreed through consensus rounds by people with HAM/TSP and asymptomatic carriers. Priority setting identified unmet needs and the most pressing concerns for people living with HTLV‐1. These included the further investigation of disease progression and its management, exploration of the psychosocial impact of an HTLV‐1 diagnosis and raising awareness and improving knowledge about HTLV‐1 in the healthcare arena and broader social world. These patient involvement workshops are informing new research proposals.

Despite receiving specialist HTLV‐1 health services widely regarded as superior in many ways to the nonspecialist services on offer elsewhere,^5^ participants identified many topics that are worthy of further investigation. Their concerns about the meaning and value of proviral load measurement echoes similar discussions in the literature. The risk of HAM/TSP has been strongly related to proviral load,^32^ but it is widely recognised that the positive predictive value is low as 50% of asymptomatic carriers have a high proviral load. Previous studies showed no association between common HAM/TSP symptoms and proviral load,^33^, ^34^, ^35^ and questions remain about the diagnostic accuracy of most biomarkers.^12^ A more recent study combining proviral load with markers of inflammation may offer a more refined risk assessment for HAM in asymptomatic carriers.^36^ Biomarker tests usually involve invasive procedures. The 10m walk test is a noninvasive measure and has the potential to be used as an outcome measure in clinical trials with people with HTLV‐1,^37^ but changes in score are difficult to interpret and demonstrate considerable variability within individuals.^12^ The identification of less invasive and more reliable biomarkers would promote better patient care and more effective ways of measuring interventions.

Workshop participants were concerned about the lack of knowledge about HTLV‐1 and reliable resources for those seeking information. Casseb^20^ has argued that HTLV‐1 should be considered a ‘neglected disease’ partly because of the lack of treatments available, but also because of the lack of information available to healthcare professionals and the public about the condition despite there being an appetite for learning about HTLV‐1.

A strong biomedical focus may make the more complex psychosocial issues associated with HTLV‐1 diagnosis and clinical management invisible to healthcare professionals. At best psychosocial needs are undervalued and at worst ignored when patients articulate a need for support and advice.^21^ Workshop participants spoke at length about the hardships they faced in their lives with HTLV‐1. They took steps to manage the impact symptoms had on their activities and participation in life. Participants felt the more complex aspects of life with HTLV‐1 were inadequately understood and not addressed by healthcare services. The workshops and recent research,^5^, ^21^ have revealed that people living with HTLV‐1 have to deal with many complex symptoms and face a number of psychological and emotional challenges that have a negative impact on HRQoL. Further research is warranted to explore and understand these issues in more depth, and to find solutions and interventions which directly address the concerns of people with HTLV‐1.

## 11 STRENGTHS AND LIMITATIONS

All participants (*n* = 27) engaged with the workshops, and most contributed across the 10 month period through to the consolidation workshops at 12 and 14 months. Several factors may contribute to the prolonged engagement of participants, many of whom, it should be acknowledged, were living lives disrupted by HAM/TSP and comorbid conditions. Participants valued and enjoyed the workshops. Meeting regularly over a period of several months assisted the establishment of relationships that allowed participants to feel comfortable enough to share their stories. Involvement at the start of the research process before any questions or topics were fixed may have helped to generate trust and commitment. The idea that participants' opinions were sought and mattered was another powerful motivator for the group. Meeting remotely helped to include participants from all over England, many of whom would have struggled to participate in person. Willingness to engage digitally may be attributed to the impact of the COVID‐19 pandemic on remote working and interaction. Considerable support was offered from the lead author who knew the participants well. These factors may have encouraged participation and persistence but may be difficult to replicate in other contexts.

## 12 CONCLUSION

This is the first report of this type of patient involvement with people with HTLV‐1 in the United Kingdom. Discovering what matters to individuals living with HTLV‐1 is a worthwhile endeavour. The patient involvement workshops were valued as an enjoyable and meaningful experience. Several avenues of investigation that have the potential to improve healthcare services and HRQoL were identified, prioritised, and shaped a research proposal that is relevant and meaningful to people living with HTLV‐1.

## AUTHOR CONTRIBUTIONS

All authors commented on, revised the work critically and iterated the paper. All authors approved this version to be published. Adine Adonis, Anne‐Marie Russell, Elizabeth Cassidy designed the study, conducted the workshops, collected data, analysed the data and agreed codes. Melanie Preston, Alpheus Shields, Sue Strachan, Sky Young, Haissata Diallo assisted in the study design, refined data evaluation, and participated in the workshop evaluation. Graham P. Taylor and Stephen Ashford provided guidance on the design and delivery of the study; commented on the manuscript, revised the work critically and iterated the paper. Graham P. Taylor will source funding for this publication.

## CONFLICT OF INTEREST STATEMENT

The authors declare no conflict of interest.

## Supporting information

Supporting information.

## Data Availability

The data that support the findings of this work are available from the corresponding author upon reasonable request.
