# Supplementary material for: Patient research priority setting partnership in human T‐cell lymphotropic virus type I
Source: Health Expect. 2023 Aug 14;26(6):2418–27. doi: 10.1111/hex.13848 (PMC10632630; doi:10.1111/hex.13848)
Supplement: Supplementary file 1 — Supporting information. [file HEX-26--s001.docx]

**Supplementary Information: Workshop Invitation**


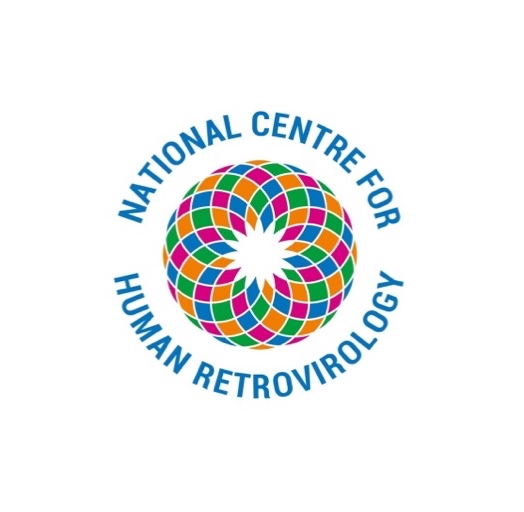

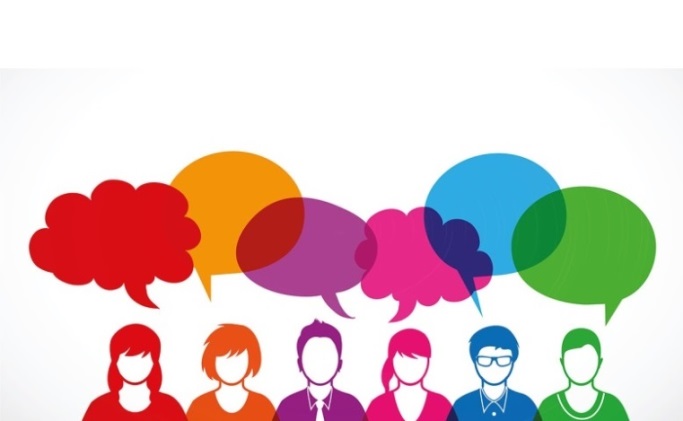


**We would like to invite you to a series of HTLV engagement events:**

**What matters most to those living with HTLV infection**?

**
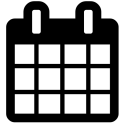
WHEN? 1^st^ Workshop 27^th^ Jan 2021 1pm – 2.30pm**

**WHERE?** [
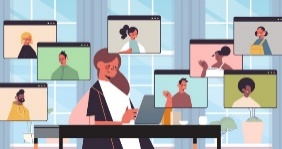
](https://www.google.co.uk/url?sa=i&url=https://www.facultyfocus.com/articles/online-education/improving-breakout-room-discussions-in-online-teaching-by-using-collaborative-documents/&psig=AOvVaw3w18UkXIHPtR1ZuhKmthLC&ust=1603287146479000&source=images&cd=vfe&ved=0CAIQjRxqFwoTCMjw45ikw-wCFQAAAAAdAAAAABAE) **Online- Zoom- or via telephone call**

**Would you like to come and find out what is happening?**

**
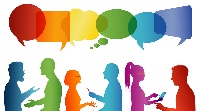
**

**Tell us when you come to clinic OR**

**
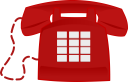
Call us on phone –020 3312 6604 OR**

**
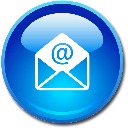
 Email: Bob Cully: noel.cully@nhs.net**
